# Effects of *Laurus nobilis* Leaf Extract on Healing of Experimentally Induced Wounds in Rabbits

**DOI:** 10.1155/2024/2889480

**Published:** 2024-10-15

**Authors:** Omar Tariq Hammoodi, Majid A. Alkhilani, Wissam Abdullah Alhayani, Waleed Al-Nuaimy, Ali A. Tala'a

**Affiliations:** ^1^Department of Surgery and Obstetrics, College of Veterinary Medicine, University of Fallujah, Anbar, Iraq; ^2^Department of Electrical Engineering and Electronics, University of Liverpool, Liverpool, UK; ^3^Department of Anatomy and Histology, College of Veterinary Medicine, University of Fallujah, Anbar, Iraq

**Keywords:** *Laurus nobilis*, leaf extract, rabbit injury, wound healing

## Abstract

Wound or injury can be defined as partial or complete separation of the skin, while the healing of the wounds is defined as the sequences of processes by which the skin heals and returns to its normal appearance. This study aims to evaluate the efficacy of *Laurus nobilis* leaf extracts on the healing of open wounds in rabbits. Twenty-four healthy rabbits were used, divided randomly into control and treated groups, each consisting of 12 rabbits. The rabbits were caused to experience circular wound defects (2.5 cm) in diameter. All animals in the control (C) group were left without treatment while those in the *Laurus nobilis* leaf extract group were treated with drops of the aqueous extract of *Laurus nobilis* in a dose of 200 mg/kg per day for 20 days. The results of macroscopic observation revealed that all animals in both groups showed equitable viability and good appetite, no mortality occurred, and no signs of infection. In the group with the *Laurus nobilis* extract, we noticed a significant improvement in wound-healing activity and a reduction in the wound area at *p* ≤ 0.05 compared with the C group. Histopathological results at the end of the study show that the thick epidermal layer covers a mass of granulation with congested blood vessels and the dermis transforms into a denser layer gradually due to the improvements of the cellularity in the C group and also revealed a well-formed skin appearance, widespread of collagen, and fibrosis within the dermis with an appearance near the normal dermis in the group treated with *Laurus nobilis* leaf extract. In conclusion, the results confirmed that using *Laurus nobilis* methanolic extract solution in a daily dose of 200 mg/kg promotes healing of open wounds in rabbits.

## 1. Introduction

The wound or injury is the complete or partial separation of the skin's layers, which occurs by different types of chemicals or physical methods [1, 2], and the normal process of wound healing for repairing the wound or injury begins at the moment the tissue is injured and consists of four sequential but overlapping phases. The first is the hemostasis phase which includes bleeding, vasoconstriction, homeostasis, and coagulation. The second is the inflammatory phase in which an inflammatory response occurs by releasing different types of mediators. The third phase is proliferative in which the production of collagen and formation of new blood vessels increases. The last phase is the remodeling phase which is characterized by refinement and increasing the tensile strength of collagen fibers and regeneration of the epithelial cells [3–5].

Various types of therapeutic methods and materials have been used to improve the route of wound healing, keep internal homeostasis, and reinstate the skin to a normal appearance [6–8]. Many plants are used in modern medicine and pharmaceutical resource for synthetic drugs [9] and to improve the treatment of skin injuries and healing of wounds [10, 11], such as the butanol extract of *Argyreia speciosa* [12], the leaf extract of *Hippophae rhamnoides* [13], and the snakehead fish extract [14].

*Laurus nobilis* belongs to the family of Lauraceae. It is an aromatic evergreen tree used in many countries around the world as a medicine against rheumatism, cough, cardiac disease, viral infection, general gastric secretion stimulant, and antiseptic [15, 16]. The essential oil and leaf extracts of the *Laurus nobilis* have been found to aid in wound healing [17]; these compounds have anti-inflammatory effects, analgesic effects, immune stimulants, antibacterial effects, and anticancer potential [18–22]. It may stimulate cell proliferation, enhance collagen production, and hasten the process of wound healing [23, 24].

This study is aimed to evaluate the efficacy of *Laurus nobilis* leaf extracts on the healing of experimentally induced open wounds in rabbits.

## 2. Materials and Methods

### 2.1. Preparation of the Methanolic Extract

*Laurus nobilis* were purchased from a local market in Iraq; the leaf was soaked in water and then dried out at room temperature and after that, the dried leaf was ground by a grinder to prepare the methanolic extract according to [25–27]. 100 g of *Laurus nobilis* leaf was used in an extraction thimble with 700 mL of 70% methanol at a temperature of 40°C for about 5–6 h, the mixture was evaporated by a rotary evaporator under vacuum at 40°C after using number 1 Whitman filter paper to exclude the methanol from the mixture, and finally, the extract was preserved in a dark glass at 4°C to be used in the treatment of the experimentally induced wounds.

### 2.2. Experimental Animals

Twenty-four healthy adult male rabbits weighed 2.0–2.5 kg, without any visible skin lesions, were used. The rabbits were kept in individual cages with 12 h of light and dark cycles in an air-conditioned environment, and they were fed on pellets and water. The study was carried out according to the Institutional Ethics Committee (IEC)'s approval and guidelines of the College of Veterinary Medicine/Fallujah University no. 23 on November 14, 2023. Thereafter, the animals were divided randomly into two equal groups of 12 rabbits in each group, the control (C) group and *Laurus nobilis* extract–treated (LET) group.

### 2.3. Surgical Technique

**Figure 1:**
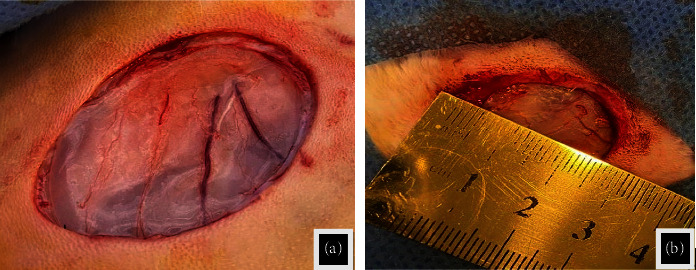
(a) and (b) The induced full-thickness circular wound: 2.5 cm in diameter in the skin.

**Figure 2:**
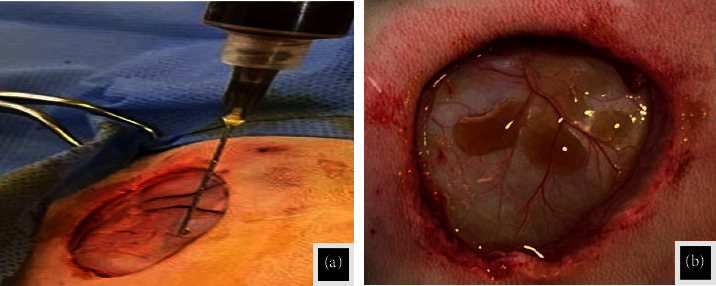
(a) and (b) The application of the *Laurus nobilis* methanolic extract on the wound.

All rabbits were caused to experience circular wound defects (2.5 cm) in diameter through the skin on the dorsal aspect of the thoracic region under aseptic preparation (Figures 1(a) and 1(b)) after giving anesthesia by injection a combination of Xylazine (5 mg/kg B.W.) and Ketamine hydrochloride (35 mg/kg B.W.) [28]. The animals in the C group were left without treatment and dressed in gauze to protect the wound from contamination. The animals of the LET group were treated by adding drops of *Laurus nobilis* methanolic extract in a dose of 200 mg/kg daily according to [29, 30] until the end of the experiment (Figures 2(a) and 2(b)). All wounds have been left without suturing to heal as an open wound for different periods until the end time of the experiment.

## 3. Assessments of Study Parameters

### 3.1. Macroscopic Observation

Daily clinical examination was performed for all animals, and all wounds of both groups were observed for the assessment of healing progression, measurement of the wound contraction, and closure by centimeter at 5, 10, 15, and 20 days postoperatively.

### 3.2. Statistical Analysis

The data were analyzed by analysis of variance (ANOVA) two-way using SPSS (Version 25), and the least significant difference (LSD) test was used to determine the differences between treatment and time.

### 3.3. Histopathological Evaluation

Animals were anesthetized, after which a full-thickness circular incision was made around the wounds of the skin. Biopsies were obtained from the healed and healthy tissue using the excision scalpel technique for microscopic examination which was performed at 5, 10, 15, and 20 days postoperatively to follow wound healing. The obtained biopsies were irrigated with normal saline, settled in a buffered formalin solution of 10% for 72 h, and then dried out using a sequence of concentrations of ethyl alcohol (70%, 80%, 90%, and 100%), and after they are cleared in xylene, they were embedded in paraffin wax and sliced to the thickness of 5–7 micrometers by using the microtome. Hematoxylin–Eosin (H&E) and Masson's trichrome stains were used to stain the slides, and after that, they were examined under a light microscope [31].

## 4. Result and Discussion

The efforts of the present study are for providing medicinal plants for use in wound healing according to macroscopic and microscopic evaluation of healing capability by using local application of *Laurus nobilis* leaf extracts on open wounds in comparison with the C group in rabbits.

### 4.1. Macroscopic Observation

All animals in both groups showed equitable viability and good appetite, no marked differences in body temperature, and no mortality occurred and tolerated the surgical procedure during the study period. Daily checking had been made for the experimentally induced skin wounds with no signs of infection, which is regarded to the aseptic technique used during surgery, postoperatively management, and follow-up on the animals. This result is consistent with [32, 33], which declared that rabbits are excellent exemplars used for wound healing.

**Figure 3:**
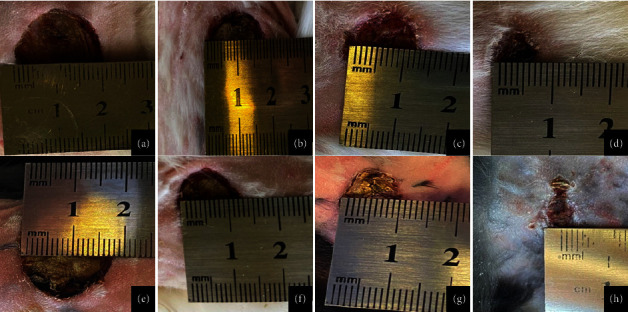
Measurement of the wound contraction by centimeter: (a–d) control group on 5^th^, 10^th^, 15^th^, and 20^th^ days postoperatively, respectively, and (e–h) LET group on 5^th^, 10^th^, 15^th^, and 20^th^ days postoperatively, respectively.

In the LET group, the reduction in the wound area is more significant than the C group at *p* ≤ 0.05 as in Figure 3 and Table 1, which shows the mean values of wound area contraction for the two groups of animals. Also, the activity of wound healing in the LET group is more significant than in the C group of animals. The significant improvement in wound healing and reduction of the wound area in the LET group compared to the C group of animals are in agreement with [30], who found that the local application of *Laurus nobilis* extracts to the wounds on rats resulted in significant contraction in the wound.

Wound contraction is considerable in the closing of the open wound, in which a force developed inside the granulation tissue pulls the peripheral edge of the skin to the center of the wound [34]. Many studies using *Laurus nobilis* in treating wounds in animals revealed a significant contraction in the wound area, granulation tissue weight, and more hydroxyproline compared with the C group [35]. Therefore, a fast contraction rate of the wound area revealed superior efficiency of the local application of *Laurus nobilis* on wound healing. The higher rate of contraction in the LET group could be associated with the stimulation of cytokines by flavonoids, terpenes, and gallic acid which are components of *Laurus nobilis* [36].

The mechanism of wound healing is contraction and re-epithelization, starting from the edge of the wound toward the center (centripetal pattern). In rats and rabbits, the course of wound healing occurs mainly by contraction rather than re-epithelization, while re-epithelization is the main process in humans [37]. The contraction of wounds in rabbits is primarily mediated by myofibroblasts [38], thus the significant activity of *Laurus nobilis* on the contraction of wounds during the periods of wound healing may be due to the angiogenesis of blood vessels and induction of mitosis activity.

### 4.2. Histopathological Evaluation of the C group

**Figure 4:**
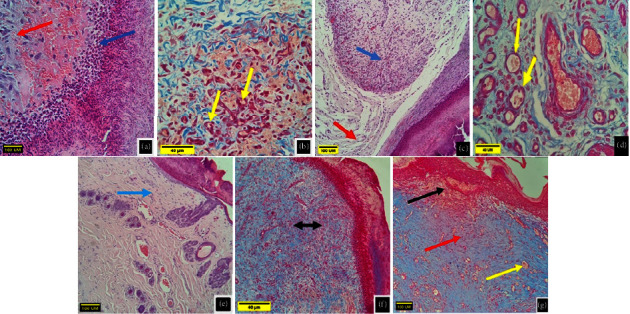
Histopathological section of the wound for the control group. (a) and (b) On the 5^th^ day postwounding shows neutrophils infiltration (blue arrow) and fibrin network formation (red arrows): wide area of hemorrhage (yellow arrow) (a): H&E X10 and (b): Masson's trichrome X40. (c) and (d) On the 10^th^ day postwounding shows granulation tissue (blue arrow) loose stroma (red arrow) and active angiogenesis (yellow arrow) (c): H&E X10 and (d): Masson's trichrome X40. (e) and (f) On the 15^th^ day postwounding shows immature granulation tissues (blue arrow) with mononuclear cells infiltration (black arrow). (e) H&E X10 and (f) Masson's trichrome X40. (g) On the 20^th^ day postwounding shows thick epidermal layer (black arrow) over the granulation tissue (red arrow) which consists of newly formed blood vessels (yellow arrow). Masson's trichrome X10.

The histopathological section of wound healing in the C group on the 5^th^ day postwounding shows infiltration of neutrophils with the presence of a fibrin network with a wide area of hemorrhage, acute inflammatory cells infiltration, and scanty collagen as shown in Figures 4(a) and 4(b). These are early signs of acute inflammation that is identified by infiltration of heterophils, edema, and vascular congestion, which indicates incomplete wound healing [39, 40].Whereas the histopathological section of the wound on the 10^th^ day postwounding showed a massive amount of granulation tissue, edema with loose stroma in the layers beneath, and angiogenesis due to the congested newly formed blood vessels as shown in Figures 4(c) and 4(d). The chronic stage of inflammation is characterized by the infiltration of mononuclear cells and fibroplasias. During the hemostasis phase and the inflammatory phase, the function of macrophages is similar to that of neutrophils and monocytes, that is, removing the bacteria and damaged tissue and producing different types of growth factors that regulate the processes of wound healing [41].

The histopathological section on the 15^th^ day postwounding in the C group shows immature granulation tissue filling the site of the lesion characterized by blood vessel formation with infiltration of mononuclear cells (Figures 4(e) and 4(f)). These results are similar to the results of [42] who found that when the collagen accumulated in the granulation tissue to produce a scar, the blood vessels would start to regress and the density of vessels would return to the baseline levels.

On the 20^th^ day postwounding, the histopathological section showed a thick epidermal layer covering a mass of granulation tissue which consisted of congested blood vessels (Figure 4(g)). While the epidermis gets thicker, it would cover the uncovered dermis, as well as the dermis transform into more dens layer gradually due to the improvements of the cellularity, deposition of the matrix, and contraction of the wound [39].

### 4.3. Histopathological Evaluation of the LET Group

**Figure 5:**
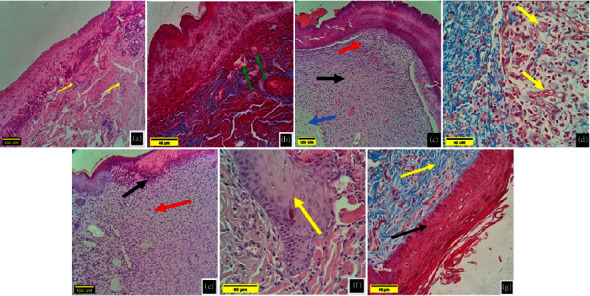
Histopathological section of the wound for the LET group. (a) and (b) On the 5^th^ day postwounding shows granulation tissue replaced most of incision area (green arrows) with congested blood vessels, neutrophils, and mononuclear cells infiltration (yellow arrow). (a): H&E X10 and (b): Masson's trichrome X40. (c) and (d) On the 10^th^ day postwounding shows large amount of well-organized granulation tissue (black arrow), mononuclear inflammatory cells (red arrow) with dense stroma (blue arrow), and sufficient vascularization (yellow arrow). (c): H&E X10 and (d): Masson's trichrome X40. (e) and (f) On the 15^th^ day postwounding shows thick epidermal area (black arrow) with mononuclear cells infiltration (red arrow) and presence of dens matures granulation tissue in the incision area (yellow arrow). (e): H&E X10 and (f): H&E X40. (g) On the 20^th^ day postwounding shows normal epidermal area (black arrow) covering mature granulation tissue (yellow arrow). Masson's trichrome X40.

The histopathological section of the samples of the LET group on the 5^th^ day postwounding showed granulation tissue filling the area of the incision with congested blood vessels, infiltration of neutrophils and mononuclear cells, edematous and loose stroma with the presence of well-defined wound scab, and collagen fiber (Figures 5(a) and 5(b)). While on the 10^th^ day postoperatively for the same group, the histopathological section showed an increased amount of granulation tissue filling the wound area, mononuclear cells, and sufficient vascularization (Figures 5(c) and 5(d)). On the 15^th^ day postoperatively, the histopathological section of the wound showed very active epithelization and significant masses of granulation tissue (Figures 5(e) and 5(f)).

On the 20^th^ day postoperatively, the histopathological section of the wound showed well-healed skin, the epidermis appeared to be close to normal, restoration of the adnexa, and the presence of widespread collagen fibers within the dermis (Figure 5(g)).

The cellularity improvement of the dermis is mainly by the proliferation of fibroblast and the deposition of the new matrix. All of the contraction of the wound, accumulation of the matrix, and remodeling lead to a decrease in the scar area of the wound [39].

The study was performed to investigate the effect of the topical application of *Laurus nobilis* leaf extract on the healing of open wounds in rabbits. In the LET group, the processes of wound healing were faster than the C group.

The early acute inflammation stage of wound healing is characterized by congestion of the blood vessels, infiltration of heterophils, and edema, while the chronic stage is characterized by fibroplasia and infiltration of mononuclear cells. During the aforementioned stage, the network of fibrin is absorbed building up a tough network of collagen in the dermis layer of the wounded area [36].

The mechanisms of wound healing are composed of overlapping phases to reinstate the skin to its normal shape, while the contraction mechanism of the wound is shrinkage of the area of the wound [43]. The process of wound healing depends on the general state of the normal tissue, the type, and intensity of the damaged tissue. The healed area of the wound is characterized by granulation tissue composed from collagen, fibroblast, and small new blood vessels. The undifferentiated mesenchymal cells on the edge of the wound transform into fibroblasts which start by migrating toward the gap of the wound with the fibrin strands that give strength and support [44]. The re-epithelialization of wound tissue starts with the migration of endothelial cells, leading to neovascularization of connective tissue [45]. *Laurus nobilis* contains antiseptic, anti-inflammatory, and antimicrobial properties due to its content of essential oils which display antimicrobial activity against different types of bacteria. When applied these essential oil to the wounds, they would help prevent infection, reduce inflammation, and promote tissue regeneration [46–48].

The faster improvement of wound reduction in the LET group is perhaps related to the stimulation of cytokine by flavonoids, terpenes, and gallic acid that are components of *Laurus nobilis* [36]. Phytochemical constituents (flavonoids) of the *Laurus nobilis* extract contain monoterpenoids which have anti-inflammatory effects that are essential for initiating the healing process and preventing further tissue damage and antimicrobial properties that aid in preventing infection in the wound [49, 50]. Also, leaf extracts of *Laurus nobilis* contain bioactive compounds that promote wound healing by stimulating blood circulation and cell proliferation, enhance collagen production that provides strength and structure of the new tissue forming at the wound site, enhance angiogenesis which is vital in delivering oxygen and nutrients to the healing wound, and accelerate the healing process. The antioxidant properties of the essential oil can protect cells from oxidative stress and promote tissue repair.

## 5. Conclusion

The study showed a clear effect of using the *Laurus nobilis* leaf extract at a dose of 200 mg/kg once a day on promoting the healing of open wounds in rabbits compared with a C group, as well as the simple method for preparation of the *Laurus nobilis* with low cost.

## Figures and Tables

**Table 1:** The mean values of the wound area contraction (cm) among the group.

Group	Day 1	Day 5	Day 10	Day 15	Day 20
Control	2.5 ± 0.06 cm A	1.93 ± 0.03 cm B	1.63 cm C a	1.27 ± 0.03 cm D a	0.73 ± 0.09 cm E a
Treated	2.5 ± 0.06 cm A	1.8 ± 0.06 cm B	1.37 ± 0.03 cm C b	0.87 ± 0.03 cm D b	0.33 ± 0.03 cm E b

*Note:* LSD = 0.2. The capital letters refer to significant differences between times (row) at *p* ≤ 0.05. The small letters refer to significant differences between control and treated (column) at *p* ≤ 0.05.

## Data Availability

The data that support the findings of this study are available from the corresponding author upon reasonable request.
